# OD22 Innovating Patient Involvement In Health Technology Assessments To Enable More Sustainable Involvement From Patient Stakeholders

**DOI:** 10.1017/S0266462324001545

**Published:** 2025-01-07

**Authors:** Heidi Livingstone, Ella Fitzpatrick, Mandy Tonkinson, Helen Crosbie, Mark Rasburn, Sally Taylor, Janine Wigmore

## Abstract

**Introduction:**

The National Institute for Health and Care Excellence (NICE) heard from small organizations how resource intensive and difficult it is for them participate in medicines health technology assessments (HTA) since the COVID-19 pandemic. To provide additional support for these organizations or to provide alternative patient input, NICE explored implementing surveys directly with patients to share with patient stakeholder organizations and NICE’s HTA medicines committees.

**Methods:**

Patient organizations and colleagues at NICE were included in the background investigation. Informal interviews were conducted with the HTA bodies in Wales and health technology colleagues in NICE about their experience of this method of patient input.

Two approaches were piloted:

(i) Developing the online questionnaire using the Summary of Information for Patients.

(ii) Developing a jointly branded questionnaire collaboratively with the patient organization and implementing a data-sharing agreement to share the raw data.

The survey was distributed by the patient organization, analyzed by NICE, and shared with the patient organization to inform their submission to NICE.

**Results:**

The results of the background investigation showed that the option to include this additional method of input could provide valuable support for patient organizations and has the potential to increase the amount and quality of patient input to an HTA committee.

Both pilots were successful in:

- Supporting patient organizations’ input into a medicines HTA
- Reducing the resources required from patient organizations.
- The second pilot added more value due to:
- Collaboration, relationship, and building trust
- Joint development of the survey
- Data sharing and potential to add to patient evidence about a disease and treatments.

**Conclusions:**

Surveys conducted directly with patients can help patient organizations participate in medicines HTAs, but they are only one element of developing more innovative and sustainable patient involvement in the process. HTA bodies need to innovate and work collaboratively with patient stakeholders to produce a menu of options for involvement so that it can be tailored to stakeholders’ resources.

